# Immature Persimmon Suppresses Amyloid Beta (Aβ) Mediated Cognitive Dysfunction via Tau Pathology in ICR Mice

**DOI:** 10.3390/cimb43010033

**Published:** 2021-06-21

**Authors:** Seul-Ki Yoo, Jong-Min Kim, Uk Lee, Jin-Yong Kang, Seon-Kyeong Park, Hye-Ju Han, Hyo-Won Park, Hyun-Jin Kim, Chul-Woo Kim, Mahn-Jo Kim, Ho-Jin Heo

**Affiliations:** 1 Division of Applied Life Science (BK21), Institute of Agriculture and Life Science, Gyeongsang National University, Jinju 52828, Korea; ysyk9412@naver.com (S.-K.Y.); myrock201@gnu.ac.kr (J.-M.K.); kangjy2132@gmail.com (J.-Y.K.); tjsrud2510@gmail.com (S.-K.P.); gksgpwn2527@naver.com (H.-J.H.); hyunjkim@gnu.ac.kr (H.-J.K.); 2 Division of Special Forest Resources, National Institute of Forest Science, Suwon 16631, Korea; rich26@korea.kr (U.L.); hyowon1119@korea.kr (H.-W.P.); futuretree@korea.kr (C.-W.K.); otttr@korea.kr (M.-J.K.)

**Keywords:** immature persimmon, *Diospyros kaki*, amyloid beta, cognitive dysfunction, tau pathway

## Abstract

This study confirmed the ameliorating effect of immature persimmon (*Diospyros kaki*) ethanolic extract (IPEE) on neuronal cytotoxicity in amyloid beta (Aβ)_1–42_-induced ICR mice. The administration of IPEE ameliorated the cognitive dysfunction in Aβ_1–42_-induced mice by improving the spatial working memory, the short-term and long-term memory functions. IPEE protected the cerebral cholinergic system, such as the acetylcholine (ACh) level and acetylcholinesterase (AChE) activity, and antioxidant system, such as the superoxide dismutase (SOD), reduced glutathione (GSH) and malondialdehyde (MDA) contents. In addition, mitochondrial dysfunction against Aβ_1–42_-induced toxicity was reduced by regulating the reactive oxygen species (ROS), mitochondrial membrane potential and ATP contents. In addition, IPEE regulated the expression levels of tau signaling, such as TNF-α, p-JNK, p-Akt, p-GSK3β, p-tau, p-NF-κB, BAX and caspase 3. Finally, gallic acid, ellagic acid and quercetin 3-O-(6″-acetyl-glucoside) were identified as the physiological compounds of IPEE using ultra-performance liquid chromatography ion mobility separation quadrupole time-of-flight/tandem mass spectrometry (UPLC IMS Q-TOF/MS^2^).

## 1. Introduction

Alzheimer’s disease (AD) is characterized by progressive degenerative changes in the cerebral cortex and hippocampus, resulting in cognitive and behavioral disturbances [1]. The main cause of AD is neuronal death caused by excessive oxidative stress and inflammatory reactions, which are considered mediators of AD [2]. In particular, the deposition of amyloid beta (Aβ) plaques and intracellular neurofibrillary tangles (NFT) is a major cause of neuronal loss [3]. Aβ is a peptide produced from the amyloid precursor protein (APP) through the activation of β-secretase and γ-secretase, and the excessive production of Aβ causes neuronal apoptosis and death [4]. In particular, the neuronal Aβ_1–42_ peptide, which plays an important role in AD pathogenesis, is not easily removed and causes apoptosis through various pathways such as inflammatory reactions because of its toxicity compared to other Aβ species [5]. In addition, Aβ damages the redox activity in the mitochondria, leading to the production of reactive oxygen species (ROS) and free radicals, and inhibits the activity of antioxidant enzymes such as superoxide dismutase (SOD) and catalase through the production of oxidative stress [6]. These amyloidogenic toxicities can cause cognitive and behavior dysfunction, psychiatric symptoms and, ultimately, AD [7].

It is also known that the generation of oxidative stress from Aβ induces the phosphorylation of tau [8]. This oxidative stress promotes an inflammatory response by increasing the expression of inflammatory cytokines such as tumor necrosis factor (TNF) and interleukin (IL-1β) [9]. Inflammatory cytokines phosphorylate c-Jun N-terminal kinases (JNK) and, ultimately, increase the abnormal phosphorylation of tau proteins, regulating the microtubule stability [10]. The tau protein, having at least 45 phosphorylation sites, is isolated from the microtubules by external stress, and aggregation of the isolated tau protein is easily accelerated due to these phosphorylation sites [11]. Eventually, the constant entanglement of tau leads to the production of NFT and neuronal death, causing cognitive impairment [8]. Therefore, it is necessary to intake antioxidants and functional food materials derived from natural resources before AD occurs to suppress the progression of disease.

Persimmon (*Diospyros kaki*) is a fruit crop grown in the tropics and subtropics, including Asian countries such as Korea, China and Japan and includes physiological compounds such as vitamins A, B1 and C; folate and chlorophyll [12]. Persimmon has an antiaging effect, preventive effect of cardiovascular disease and anticancer effects based on its high antioxidant effect derived from a variety of phytochemicals, such as gallic acid, tannins and catechins [13,14,15]. In particular, it contains a large amount of tannins, which act similar to fibrin, and protects against the onset of metabolic diseases by improving the cholesterol levels [16]. Although persimmons have various physiological activities, the strength of persimmon fruit is lowered before harvesting, and thus, it is prone to damage during distribution and storage [17]. Additionally, there is a large amount of persimmon fruit drops before harvest caused by a combination of various factors [18]. Since fruit drops represent more than 90% of the total production, economic and environmental problems arise. Thus, measures such as the development of processed products are needed. Therefore, in this study, to increase the value added of the reuse of discarded immature persimmons, we investigated the improvement effect of immature persimmon (*Diospyros kaki*) ethanolic extract on Aβ_1–42_-induced ICR mice and evaluated the industrial availability and potential as a raw material for functional food ingredients.

## 2. Materials and Methods

### 2.1. Chemicals

Amyloid beta (Aβ)_1__–42_, hydroxylamine hydrochloride, sodium hydroxide, hydrochloride, ferric (III) chloride hexahydrate, metaphosphoric acid, o-phthaldialdehyde, phosphoric acid, thiobarbituric acid (TBA), mannitol, sucrose, bovine serum albumin (BSA), HEPES sodium salt, 3,12-bis(carboxymethyl)-6,9-dioxa-3,12-diazatetradecanedioic acid (EGTA), 2′,7′-dichlorofluorescein diacetate (DCF-DA), digitonin, potassium chloride, potassium phosphate, HEPES, magnesium chloride, pyruvic acid, malic acid, JC-1, protease inhibitor, polyvinylidene difluoride (PVDF) membrane, sodium azide and all other chemicals were purchased from Millipore (Billerica, MA, USA). Primary antibodies such as phosphorylated c-Jun N terminal kinase (p-JNK) (sc-6254), glycogen synthase kinase 3 beta (GSK3β) (sc-9166), phosphorylated-tau (p-tau) (sc-12952), cytochrome C (sc-13560), caspase 3 (sc-56053) and β-actin (sc-69879) were purchased from Santa Cruz Biotechnology (Santa Cruz, CA, USA). Other primary antibodies such as tumor necrosis factor-alpha (TNF-α) (3707S), phosphorylated-nuclear factor kappa-light-chain-enhanced of activated B cells (p-NF-κB) (#3031), phosphorylated-protein kinase (p-Akt) (#9271), BAX (#2772) and secondary antibodies such as anti-rabbit (7074S) and anti-mouse (7076S) were purchased from Cell Signaling Technology (Danvers, MA, USA).

### 2.2. Sample Preparation

The immature persimmon (*Diospyros kaki*) used was harvested in July in Sangju-gun (Sangjudungsi cultivar) and verified by the National Institute of Forest Science (Suwon, Korea). The pulp of immature persimmons was extracted with 50-fold 20% ethanol at 40 °C for 2 h. The extract was filtered through No. 2 filter paper (Whatman PLC, Kent, UK) and concentrated using a vacuum rotary evaporator (N-N Series Eyela Co., Tokyo, Japan). The concentrate was lyophilized and stored at −20 °C for the experiments.

### 2.3. In Vivo Experimental Design

Institute of Cancer Research (ICR) mice, widely used in various research as an evaluation of toxicology, pharmacology and development of pharmaceutical products for decades (male, 4 weeks old), were supplied by Samtako (Osan, Korea) [19]. The mice were housed two and three per cage and divided into four groups. Experimental conditions were maintained at standard laboratory condition humidity and temperature (12-h light–dark cycle, 55% humidity and 22 ± 2 °C temperature). Immature persimmon ethanolic extract (IPEE) samples were dissolved in distilled water (*w*/*v*) and orally fed once a day for three weeks (50 and 100-mg/kg body weight for the IPEE 50 and IPEE 100 groups, respectively). The intake concentration of the sample was decided based on previous research and referred to various studies [20,21,22,23]. After 3 weeks, 410-pM Aβ_1–42_ was intracerebroventricularly (icv) injected at bregma with a 25-μL Hamilton micro syringe fitted with a 26-gauge needle. The control group was injected with 0.85% sodium chloride solution without Aβ_1–42_ [24]. All animal experiments were approved by the Animal Care and Use Committee of Gyeongsang National University (certificate: GNU-181019-M0054 and 19 September 2018), and all experiments were conducted according to the provisions of the Policy of the Ethical Committee of the Ministry of Health and Welfare, Republic of Korea.

### 2.4. In Vivo Behavior Tests

To evaluate spontaneous alternations and spatial working memory, a Y-maze test was performed over 3 days after the Aβ injection. The Y-maze was composed of black plastic with three arms that were 33 cm long, 15 cm high and 10 cm wide. The mice were placed in the designated arm, and the behavior patterns of their explorations were recorded for 8 min by a smart 3.0 video tracking system (SMART v3.0, Panlab SL, Barcelona, Spain). When the mice serially entered three different arms, it was considered a successive alternation behavior [25].

To investigate short-term memory functions, a passive avoidance test was conducted over 2 days. The passive avoidance box consisted of separated light and dark zones. The mice were located in the light zone for 2 min. When a mouse entered the dark zone after a guillotine door was opened, the guillotine door was closed, and an electric shock (0.5 mA, 1 s) was delivered. After 24 h, each mouse was located in the light zone again, and the step-through latency to enter the dark zone was recorded (maximum 300 s) [26].

To examine long-term learning and spatial memory function, a Morris water maze test was conducted. The experimental pool consisted of a stainless-steel circular tank (90 cm in diameter) containing water at 20 ± 2 °C. The Morris water maze pool was divided into quadrants (E, S, N and W zones) with marked visual clues in opaque water with squid ink (Cebesa, Valencia, Spain). A platform was positioned in the middle of the W zone and relocated during the test. The latency time was recorded over 60 s by a smart 3.0 video tracking system (SMART v3.0, Panlab SL). In a probe test, the mice swam freely in the water tank without the platform for 60 s, and the retention time in the W zone was recorded [27].

### 2.5. Preparation of Tissue

After an in vivo test, collected brain tissue was homogenized using a bullet blender (Next Advance Inc., Averill Park, NY, USA) with 10-fold volumes of phosphate-buffered saline (PBS, pH 7.4) and phosphate buffer (pH 6.0). The protein concentration was measured using the Bradford protein assay [28].

### 2.6. Acetylcholine (ACh) Contents and Acetylcholinesterase (AChE) Activity

To assess the acetylcholine (ACh) content, the brain tissue was homogenized with 10-fold phosphate buffer. The homogenates were centrifuged at 12,000× *g* for 30 min at 4 °C. The supernatant was mixed with hydroxylamine reagent containing 0.1-M hydrochloride and 3.5-N sodium hydroxide. After mixing, 0.5-N hydrochloride and 0.3-M ferric (III) chloride hexahydrate were added. ACh contents were assessed using a microplate reader (Epoch 2, BioTek Instruments, Inc., Winooski, VT, USA) at 540 nm [29].

To confirm the acetylcholinesterase (AChE) activity, the supernatant was mixed with 50-mM sodium phosphate buffer, and the assay was started by adding Ellman’s reaction mixture. The results were assessed by a microplate reader (Epoch 2, BioTek Instruments, Inc.) at 405 nm [30].

### 2.7. Measurement of Antioxidant Biochemical

To measure the superoxide dismutase (SOD) contents, the homogenates with 10-fold phosphate buffer were centrifuged at 400× *g* for 10 min at 4 °C. The supernatant was mixed with the working solution and enzyme according to the instructions of the manufacturer of the SOD determination kit (Sigma-Aldrich Chemical Co., St. Louis, MO, USA), and the mixture was incubated at 37 °C for 20 min. The SOD contents were measured by a microplate reader (Epoch 2, BioTek Instruments, Inc.) at 450 nm, and the SOD contents were presented as units/mg of protein.

To measure the contents of reduced glutathione (GSH), the homogenates were centrifuged at 400× *g* for 10 min at 4 °C. The obtained supernatant and 30% metaphosphoric acid were mixed and centrifuged at 2000× *g* for 2 min at 4 °C. The supernatant was reacted with 0.26-M Tris-HCl buffer (pH 7.8), 0.65-N NaOH and 1-mg/mL o-phthaldialdehyde, and this mixture was incubated in the dark for 15 min at room temperature. The reduced GSH contents were measured by a fluorescence microplate reader (Infinite 200, Infinite F200, Tecan, Mannedorf, Switzerland) from 320 nm (excitation filter) to 420 nm (emission filter). The measured contents were shown as a value relative to the reduced GSH standard curve [31].

To measure the MDA contents, homogenates were centrifuged at 2500× *g* for 10 min at 4 °C. The obtained supernatant was mixed with a 1% phosphoric acid and 0.67% TBA solution. The mixtures were combined and incubated for 1 h at 95 °C in a water bath. The MDA contents were measured by the microplate spectrometer at 532 nm and presented as nmole/mg of protein [32].

### 2.8. Measurement of Mitochondrial Activity

The brain tissue was homogenized using mitochondrial isolation (MI) buffer, including 215-mM mannitol, 75-mM sucrose, 0.1% BSA and 20-mM HEPES sodium salt (pH 7.2), and 1-mM EGTA was added. The homogenate was centrifuged at 1300× *g* for 10 min at 4 °C, and the supernatant was centrifuged at 3000× *g* for 10 min at 4 °C. Afterward, the pellet was mixed with 0.1% digitonin, and the mixture and MI buffer containing 1-mM EGTA was added and centrifuged at 13,000× *g* for 15 min at 4 °C. Finally, the obtained pellet and MI buffer were mixed and centrifuged again at 10,000× *g* for 10 min at 4 °C. The final pellet was resuspended in MI buffer to perform the mitochondrial experiment.

The ROS levels in the mitochondria were measured by the DCF-DA assay. The isolated mitochondria were reacted with DCF-DA for 20 min with respiration buffer (125-mM potassium chloride, 2-mM potassium phosphate, 20-mM HEPES, 1-mM magnesium chloride and 500-µM EGTA). The DCF fluorescence intensity was measured at 488 nm (excitation wavelength) and 535 nm (emission wavelength) by a fluorescence microplate reader (Infinite 200, Tecan Co., Männedorf, Switzerland) [32].

The level of mitochondrial membrane potential was measured using the fluorescent JC-1 with a separated mitochondrial extract. The mitochondria extract reacted with the MI buffer containing 5-mM pyruvic acid, and 2.5-mM malic acid were added to the wells of a black 96-well plate followed by the addition of 1-μM JC-1. The reaction solution was incubated at room temperature for 20 min in the dark, and then, the fluorescence was detected using a fluorescence microplate reader (Infinite 200, Tecan Co.) at 530 nm (excitation filter) and 590 nm (emission filter) [32].

The ATP contents were measured using an ATP bioluminescence assay kit (Sigma-Aldrich Chemical Co.) following the manufacturer’s instructions. The separated mitochondria were determined through the oxidation value of luciferase, and in the case of it being catalyzed by luciferase, it was determined by using the value of oxidized luciferin. The ATP contents were calculated according to a standard curve, which appeared as the nmole/mg of protein.

### 2.9. Protein Expression Evaluation

The brain tissue was homogenized in RIPA lysis buffer (Thermo Fisher Scientific, Rockford, IL, USA) containing 1% protease inhibitor and centrifuged at 13,000× *g* for 10 min. The protein contents in the supernatant were relatively quantified using a Bradford protein assay. Afterward, the protein in the samples was separated by sodium dodecyl sulfate-polyacrylamide gel electrophoresis (SDS-PAGE) and then transferred to a polyvinylidene difluoride (PVDF) membrane (Millipore, Billerica, MA, USA). The membranes were blocked with 5% skim milk and then placed in diluted primary antibody (1:1000) solution overnight and incubated with diluted secondary antibody (1:10,000). Immunoreactive bands were detected using an image analyzer (iBright™ CL1000 instrument, Invitrogen, Carlsbad, CA, USA). The density of the band was measured with ImageJ Software (National Institutes of Health, Bethesda, MD, USA).

### 2.10. Bioactive Compound Analysis

The physiological compounds were identified using UPLC IMS Q-TOF/MS^2^ (Acquity UPLC Class 1, Waters Corp., Milford, MA, USA). Separation of the physiological compounds was performed on an Acquity UPLC BEH C_18_ column (2.1 mm × 100 mm, 1.7 μm particle size; Thermo, Waltham, MA, USA) with a flow rate of 0.4 mL/min. The mobile phases consisted of solvent A (0.1% formic acid in distilled water) and solvent B (acetonitrile) during analysis. The gradient conditions were as follows: a gradient elution of 0% B at 0–0.5 min, 0–100% B at 0.5–8 min, 100% B at 8–8.5 min, 100–0% B at 8.5–10 min and 0% B at 10–11 min. The UPLC-QTOF/MS^2^ system was analyzed using MS^2^ data analysis software (Waters Masslynx TM. Waters Corp.).

### 2.11. Statistical Analysis

All experimental data were presented as the mean ± standard deviation (SD). Statistically significant differences between the groups were indicated by one-way analysis of variance (ANOVA). Significant differences in each data were found using Duncan’s new multi-range test (*p* < 0.05) with the SAS program (version 9.4, SAS Institute Inc., Cary, NC, USA), and different small letters represented statistical differences.

## 3. Results

### 3.1. In Vivo Behavior Tests

The accumulation of Aβ stimulates oxidative stress and inflammatory responses, resulting in damage to the hippocampus, amygdala, thalamus and cerebellum, leading to behavioral and memory impairments. Therefore, a behavioral evaluation reflects the functional impairment in the early stages of AD as an index for judging the learning and cognitive dysfunctions [33].

**Figure 1:**
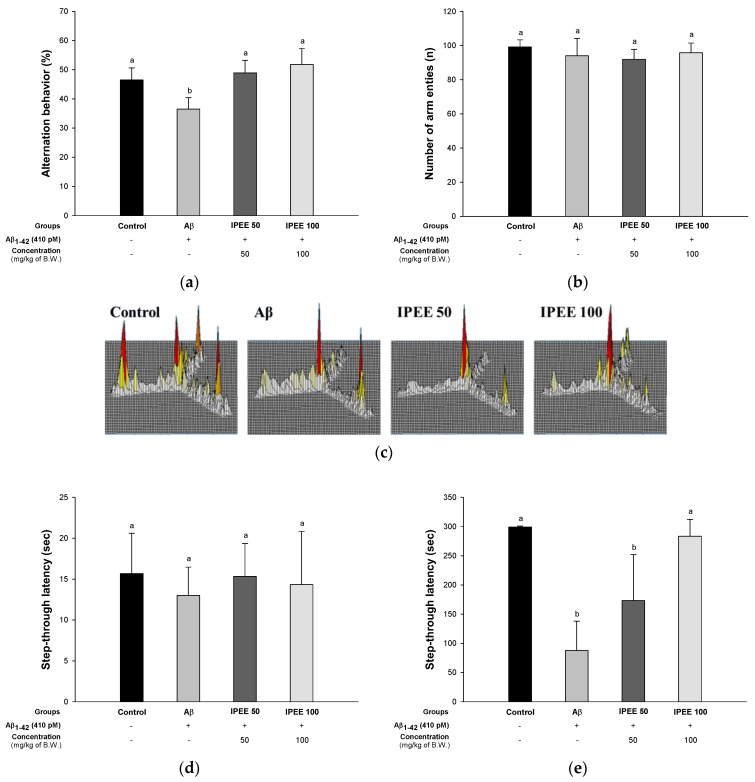

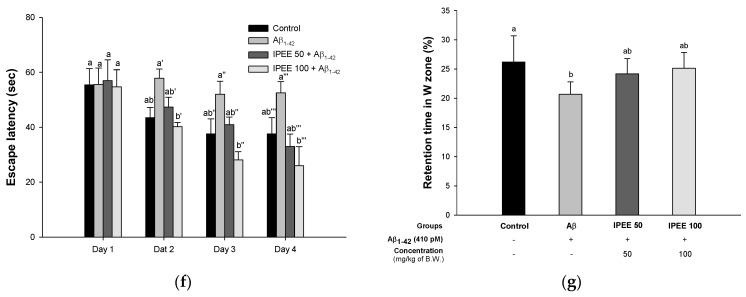
Protective effect of immature persimmon (*Diospyros kaki*) ethanolic extract (IPEE) on Y-maze, passive avoidance and Morris water maze tests in Aβ-induced mice. (**a**) Spontaneous alternation behavior in the Y-maze, (**b**) number of arm entries in the Y-maze, (**c**) tracing path in the Y-maze, (**d**) passive avoidance on the first day, (**e**) test trial on the second day in the passive avoidance test, (**f**) escape latency in the training trial in the Morris water maze test and (**g**) retention time in the W zone of the probe trial in the Morris water maze test. Results shown are the mean ± SD (*n* = 7). Data shown were statistically considered at *p* < 0.05, and different small letters in the graph represent statistical differences.

The Y-maze test is a method to evaluate the spatial cognitive function and the networks between the hippocampus, prefrontal cortex, basal forebrain and cerebellum, primarily using the rodent’s instinct to explore new environments [25]. To evaluate the protective effect of IPEE on Aβ-induced memory and learning impairment, behavior tests were performed (Figure 1). A Y-maze test was conducted to measure the working memory using the habits of rodents exploring a new pathway. The results of the Y-maze test are presented in Figure 1A–C. The alternation behaviors of the IPEE groups (48.91% ± 4.30% and 51.78% ± 78%) increased more than the Aβ group (36.47% ± 3.91%) (Figure 1A). The results of number of arm entries were not significant (Figure 1B). Additionally, it was confirmed that the behavior of the Aβ group was irregular compared to the control group in the result of moving accumulation (Figure 1C). Additionally, as the treatment concentration of IPEE increased, the working memory was significantly improved by reducing the irregular movements.

Passive avoidance learning is a method of measuring the sensitivity to parahippocampal lesions and the modulation of cholinergic mechanisms related to the thalamus, amygdala, hippocampus and various cortical areas [26]. To assess the short-term memory of mice, a passive avoidance test was conducted (Figure 1D,E). The latency time of all groups on the first day was not significant (Figure 1D). The latency time of the IPEE groups (173.33 ± 78.68 s and 283.33 ± 28.87 s) on the second day increased more than the Aβ group (87.67 ± 50.21 s) (Figure 1E).

The Morris water maze test, as a spatial test different from the passive avoidance test, is generally related to the hippocampal NMDA receptor function and long-term potentiation, so it is an index used to evaluate spatial and long-term memory learning functions [27]. To evaluate the long-term learning and memory abilities of mice, the Morris water maze test was conducted (Figure 1F–H). In the hidden test trial on the fourth day, the escape latency of the IPEE groups (32.97 ± 4.53 s and 25.95 ± 6.97 s) was reduced more than the Aβ group (52.52 ± 4.07 s) (Figure 1F). The retention time in the proven trial test of the IPEE groups (24.19% ± 2.59% and 25.15% ± 2.70%) decreased compared to the Aβ group (20.67% ± 2.13%) (Figure 1G).

### 3.2. Acetylcholine (ACh) Contents and Acetylcholinesterase (AChE) Activity

**Figure 2:**
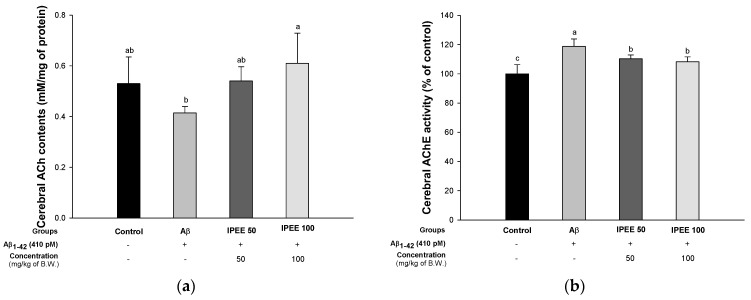
Protective effect of IPEE on Aβ-induced cholinergic dysfunction in mice cerebral tissue. (**a**) ACh contents and (**b**) AChE activity. Results shown are the mean ± SD (*n* = 7). Data shown were statistically considered at *p* < 0.05, and different small letters in the graph represent statistical differences.

ACh, one of the neurotransmitters, affects the memory by regulating cholinergic synaptic signals in the brain [34]. ACh is degraded by AChE in the cholinergic synaptic cleft, and the degradation of ACh due to excessive AChE activity indicates the impairment of cholinergic neurotransmission and is associated with cognitive dysfunction [35]. To evaluate the ameliorating effect on the cholinergic system, the activity of ACh and AChE was investigated (Figure 2). The ACh content of the Aβ group (0.41 ± 0.03 mM/mg of protein) significantly decreased compared to the control group (0.53 ± 0.10 mM/mg of protein) (Figure 2A). However, the IPEE groups (0.54 ± 0.06 mM/mg of protein and 0.61 ± 0.12 mM/mg of protein) showed a remarkable increase of ACh. In addition, the AChE activity of the Aβ group (118.84% ± 5.06%) was significantly activated compared to the control group (100.00% ± 6.28%) (Figure 2B). However, the AChE activity of the IPEE groups (110.24% ± 2.65% and 108.24% ± 3.39%) was considerably inhibited.

### 3.3. Measurement of Antioxidant Activity

**Figure 3:**
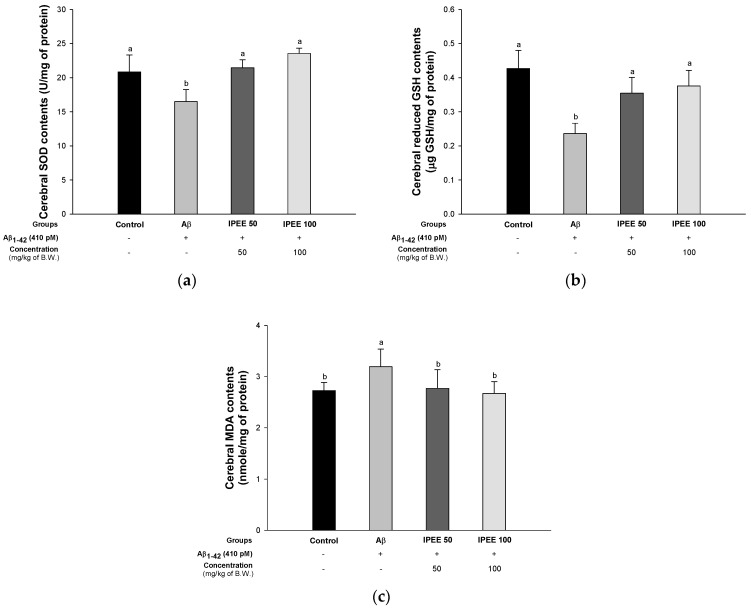
Protective effect of IPEE on Aβ-induced biochemical changes related with the antioxidant system in mice cerebral tissue. (**a**) SOD contents, (**b**) reduced GSH contents and (**c**) MDA contents in mice brain tissues. Results shown are the mean ± SD (*n* = 7). Data shown were statistically considered at *p* < 0.05, and different small letters in the graph represent statistical differences.

The antioxidant system in the brain tissue is responsible for breaking down superoxide and hydrogen peroxide into nontoxic compounds such as oxygen and water [36]. Oxidative stress caused by Aβ reduces antioxidant enzymes such as SOD, glutathione peroxidase (GPx) and GSH in the brain tissue. In particular, the hippocampus tends to be easily affected by Aβ [37]. To investigate the improvement effect of IPEE in the antioxidant system, the SOD, glutathione (GSH) and malondialdehyde (MDA) contents were measured (Figure 3). The SOD content of the Aβ group (16.50 ± 1.76 U/mg of protein) was reduced compared to the control group (20.85 ± 2.49 U/mg of protein) (Figure 3A). However, the IPEE groups (21.46 ± 1.17 U/mg of protein and 23.55 ± 0.78 U/mg of protein) showed increased SOD contents. The reduced GSH content of the Aβ group (0.24 ± 0.03 μg GSH/mg of protein) was decreased compared to the control group (0.43 ± 0.05-μg GSH/mg of protein) (Figure 3B). However, the IPEE groups (0.35 ± 0.05-μg GSH/mg of protein and 0.38 ± 0.05-μg GSH/mg of protein) showed increased reduced GSH contents. The MDA content of the Aβ group (3.19 ± 0.34 nmole/mg of protein) was excessively produced compared to the control group (2.73 ± 0.16 nmole/mg of protein) (Figure 3C). However, the IPEE groups (2.77 ± 0.36 nmole/mg of protein and 2.67 ± 0.23 nmole/mg of protein) suppressed the increase in MDA contents.

### 3.4. Measurement of Mitochondrial Activity

Damaged mitochondria exhibit decreased electron transport efficiency, ATP biosynthesis abnormality and oxidative stress production, resulting in inadequate energy production and abnormalities in calcium homeostasis, leading to mitochondrial membrane potential disruption [38]. This damage activates the apoptosis pathway in neurons through the release of cytochrome c present inside the mitochondria. Therefore, an increase in ROS and a decrease in mitochondrial membrane potential from damaged mitochondria can induce apoptosis [39].

**Figure 4:**
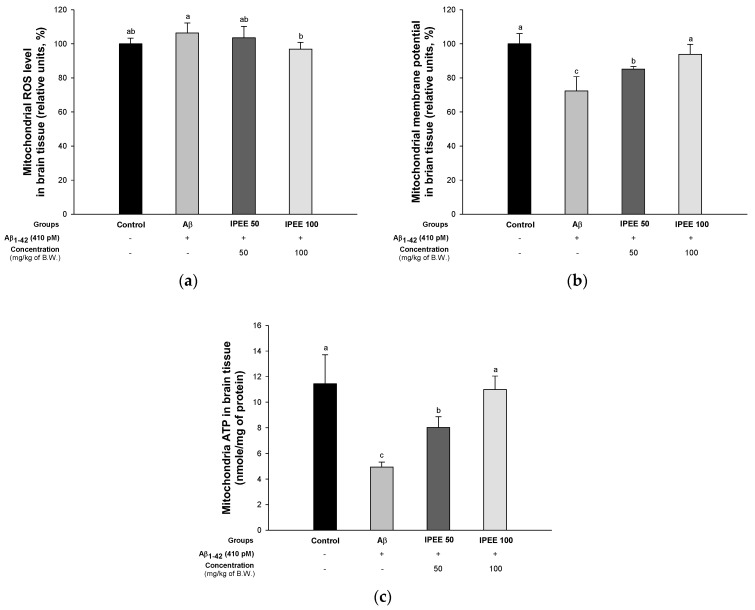
Protective effect of IPEE on Aβ-induced mitochondrial dysfunction in mitochondria from mice cerebral tissue. (**a**) ROS levels, (**b**) mitochondrial membrane potential levels and (**c**) ATP contents of the mitochondria in mice brain tissues. Results shown are the mean ± SD (*n* = 5). Data shown were statistically considered at *p* < 0.05, and different small letters in the graph represent statistical differences.

To estimate the ameliorating effect of IPEE in the neuronal mitochondrial function, the ROS contents and mitochondrial membrane potential were measured (Figure 4). DCF-DA is a method used to measure the intracellular ROS level using a fluorescence while being changed to DCF by the esterase [40]. The ROS content of the Aβ group (106.33% ± 5.86%) was increased compared to the control group (100.00% ± 3.35%) (Figure 4A). However, the IPEE groups (103.53% ± 6.70% and 96.88% ± 4.00%) showed reduced ROS contents. The mitochondrial membrane potential of the Aβ group (72.32% ± 8.41%) was decreased compared to the control group (100.00% ± 5.96%) (Figure 4B). The IPEE groups (85.14% ± 1.48% and 93.84% ± 5.82%) had significantly increased mitochondrial membrane potential levels. The mitochondrial ATP content of the Aβ group (4.95 ± 0.38 nmole/mg of protein) was considerably reduced compared to the control group (11.44 ± 2.28 nmole/mg of protein) (Figure 4C). However, the IPEE groups (8.02 ± 0.84 nmole/mg of protein and 11.00 ± 1.05 nmole/mg of protein) had increased mitochondrial ATP contents.

### 3.5. Protein Expression Evaluation

**Figure 5:**
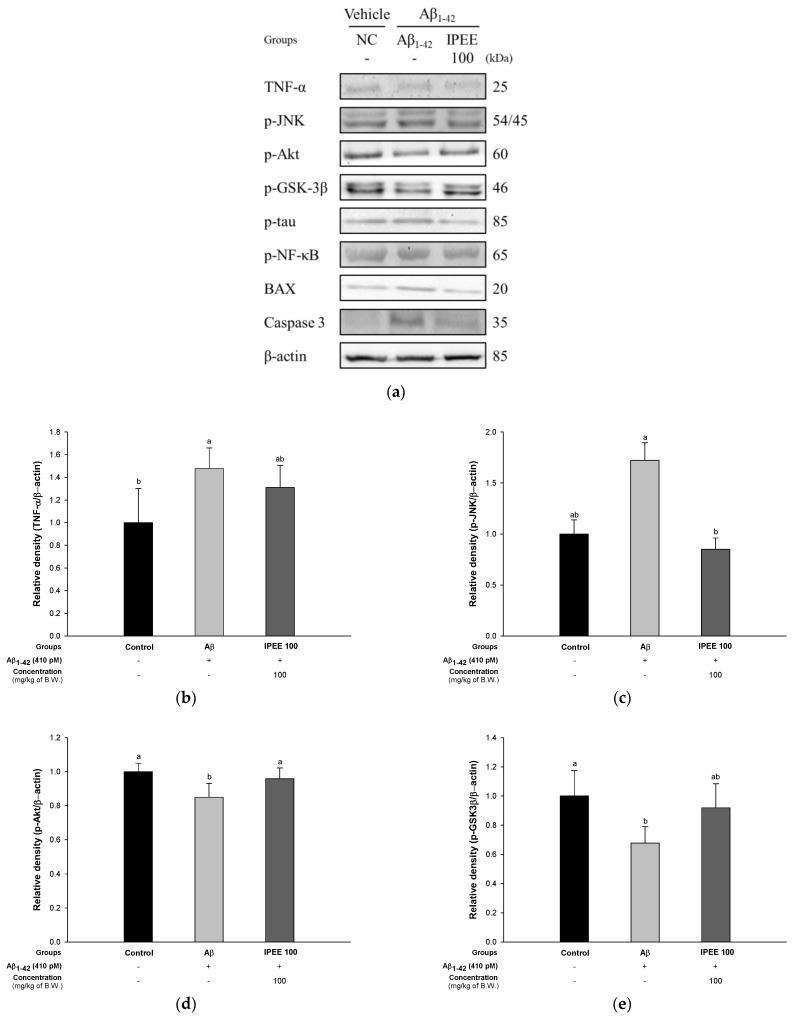

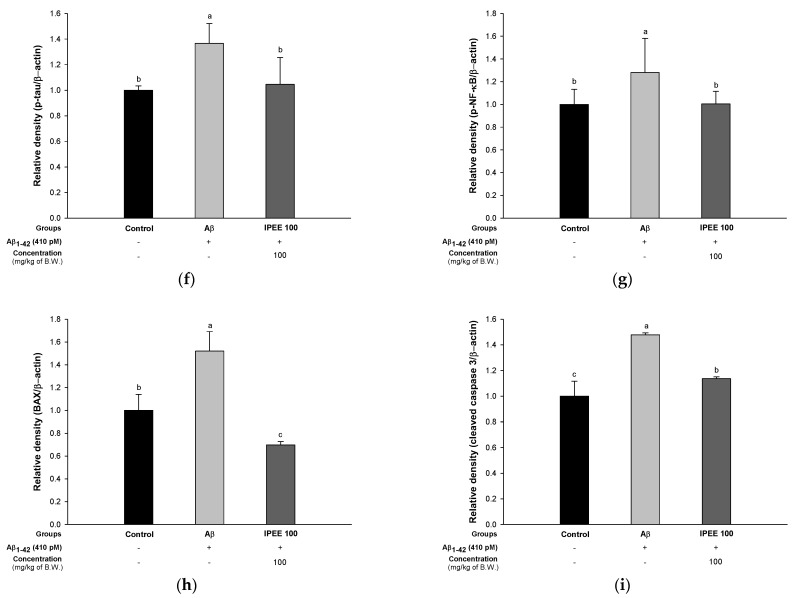
Protective effect of IPEE on the tau pathway in mice cerebral tissue. (**a**) Representative Western blots for the total protein and expression of TNF-α, p-JNK, p-Akt, p-GSK3β, p-tau, p-NF-κB and β-actin. Protein expression levels of TNF-α (**b**), p-JNK (**c**), p-Akt (**d**), p-GSK3β (**e**), p-tau (**f**), p-NF-κB (**g**), BAX (**h**) and cleaved caspase 3 (**i**) normalized to β-actin. Results shown are the mean ± SD (*n* = 3). Data shown were statistically considered at *p* < 0.05, and different small letters in the graph represent statistical differences.

To evaluate the regulatory effect of IPEE via the tau pathway, the protein expressions of TNF-α, p-JNK, p-Akt, p-GSK3β, p-tau and p-NF-κB were measured (Figure 5). The expression of TNF-α (147.66% ± 18.29%) and p-JNK (172.17% ± 17.17%) in the Aβ group was significantly upregulated compared to the control group (Figure 5B,C). The IPEE 100 group statistically downregulated the TNF-α (130.96% ± 19.52%) and p-JNK (85.10% ± 11.00%) expression levels compared to the Aβ group. The expression of p-Akt (84.99% ± 8.16%) and p-GSK3β (67.78% ± 11.16%) in the Aβ group was significantly reduced compared to the control group (Figure 5D,E). The IPEE 100 group statistically increased the expression level of p-Akt (95.83% ± 6.38%) and p-GSK3β (91.87% ± 16.54%) compared to the Aβ group. The expression of p-tau (136.66% ± 15.49%) and p-NF-κB (128.08% ± 30.01%) in the Aβ group was significantly increased compared to the control group (Figure 5F,G). The expression of BAX (152.05% ± 17.14%) and caspase 3 (147.81% ± 1.60%) in the Aβ group was significantly increased compared to the control group (Figure 5H,I). The IPEE 100 group statistically downregulated the expression of p-tau (104.55% ± 21.00%), p-NF-κB (100.44% ± 11.08%), BAX (69.82% ± 3.03%) and caspase 3 (113.64% ± 1.50%) compared to the Aβ group.

### 3.6. Bioactive Compound Analysis

**Figure 6:**
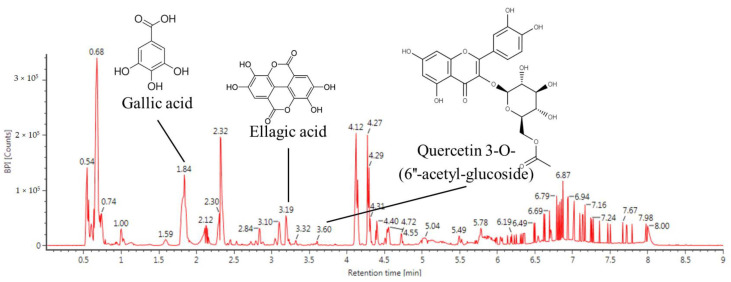
Ultra-performance liquid chromatography–ion mobility separation–quadrupole time-of-flight/tandem mass spectrometry (UPLC Q-TOF/MS^2^) chromatographic profile of IPEE.

The major physiological compounds of IPEE were qualitatively identified by an ultra-performance liquid chromatography ion mobility separation quadrupole time-of-flight/tandem mass spectrometry (UPLC IMS Q-TOF/MS^2^) analysis (Table 1 and Figure 6). The MS chromatogram obtained in the negative ion mode was presented as compound **1** (parent ion, 169 *m/z*; RT, 1.84 min), compound **2** (parent ion, 300 *m/z*; RT, 3.19 min) and compound **3** (parent ion, 505 *m/z*; RT, 3.60 min). The compounds identified were gallic acid (compound **1**, PubChem CID: 370), ellagic acid (compound **2**, PubChem CID: 5281855) and quercetin 3-O-(6″-acetyl-glucoside) (compound **3**, PubChem CID: 10006384), respectively.

## 4. Discussion

AD associated with neuronal death is one of the common neurodegenerative diseases that gradually causes a loss of memory and cognitive dysfunction among the elderly. The pathological characteristics of AD are Aβ plaque production, the aggregation of tau proteins and loss of cerebral neurons [41]. In particular, hippocampal neuronal cells have a structure that is easily damaged by Aβ [1]. Aβ induces the accumulation of oxidative stress, lipid peroxide, mitochondrial and DNA damage and the apoptosis cascade in cerebral tissue [42]. This neuronal cell death causes impaired cognitive functions, which ultimately lead to AD [43]. Therefore, this study investigated whether the consumption of IPEE can ameliorate cognitive dysfunction in an Aβ-induced deficit of antioxidant and cholinergic systems and mitochondrial dysfunction in brain tissue.

The most remarkable features of Alzheimer’s patients are the formation of plaque and oxidative stress due to the accumulation of amyloids and the neuronal inflammatory reaction in the hippocampus, amygdala and thalamus [44]. Persistent damage to neuronal cells leads to cognitive and memory impairment [1]. Thus, it is important to assess cognitive dysfunction through a behavioral assessment early in AD [45]. Therefore, the ameliorating effect on cognitive dysfunction was assessed by conducting the Y-maze, passive avoidance and Morris water maze tests (Figure 1). Immature persimmons improved the spatial cognitive ability and learning ability by downregulating the phosphorylation of the c-Jun N-terminal kinase (p-JNK) and phosphorylated insulin receptor substrate-1 (p-IRS-1) in a diabetic mice model induced by a high-fat diet for 16 weeks [46]. Ellagic acid, one of the physiological compounds in persimmons, significantly impeded the cholinergic dysfunction associated with Alzheimer’s dementia through chronically scopolamine-induced Wister rats [47]. In addition, the administration of oligomers isolated from persimmon fruits protected against cognitive dysfunction by increasing the phosphorylation of vascular endothelial growth factor receptor (VEGFR)-2 in the hippocampal CA3, hypothalamus and choroid plexus [48]. Based on these results, it is suggested that IPEE indirectly has a protective effect against various brain tissue damages, including the hippocampus, amygdala, thalamus and cerebellum, induced by Aβ. IPEE is expected to be used as a material that could improve cognitive function.

The accumulation of Aβ causes damage to the cholinergic system, which plays a role in the signaling of cognitive function [1]. The generation of Aβ plaques leads to the death of cholinergic cells. In this process, AChE bound to the cell membrane accelerates the decomposition of ACh by elution to cytosol [49]. In addition, free AChE promotes the formation of the Aβ-AChE complex by binding to the increased Aβ peptide. This complex has been reported to accelerate damage to the cholinergic system, because it has a higher toxicity than general Aβ [50]. Consequently, the inhibition of cholinergic system damage and suppression of cholinergic cell death play an important role in improving cognitive function. Therefore, the ameliorating effect on the cholinergic system was confirmed to assess the ACh content and inhibitory effect of AChE (Figure 2). According to a previous study, the ethyl acetate fraction of immature persimmon peel containing quercetin and quercetin aglycone such as quercetin 3-O-galactoside, quercetin-3-O-galactoside-2′-O-gallate and quercetin-3-O-glucoside inhibits the activity of AChE and butyrylcholinesterase (BuChE) [51]. In addition, the gallic acid contained in IPEE improves the synaptic strength and decreases the size of Aβ plaques in cerebral tissues through an improvement effect on synaptic damage induced by Aβ peptides [52]. In addition, the ACh content was increased by the inhibitory effect of AChE, which showed improvement in memory and learning ability [53]. Therefore, the improvement effect of IPEE protects the cholinergic system with the reduction of the Aβ plaque content and the inhibitory activity of AChE and could have potential as a functional food for the prevention or treatment of AD.

Oxidative stress generated in the early onset of AD plays a key role in the development of the disease [54]. Aβ_1-40_ and Aβ_1–42_ promote the production of oxidation products of proteins, lipids and nucleic acids in AD patients [55]. In particular, it was confirmed that the oxidation of neuronal components occurs in the brain regions rich in Aβ, such as the hippocampus and cerebral cortex [56]. This stress promotes the production of 4-HNE (4-hydroxynonenal) and peroxynitrite (ONOO–), which leads to neuronal damage [57]. However, the cell membrane of brain tissue is vulnerable to external stress due to the relatively insufficient antioxidant enzyme system [58]. Therefore, the protective effect on the antioxidant system was evaluated by administrating IPEE, which ameliorated the SOD and reduced GSH contents and inhibited MDA production (Figure 3). According to a previous study, the administration of persimmons decreased the lipid peroxidation and inhibited the reduced SOD and GSH contents [59]. In addition, the lipid peroxidation and antioxidant systems were improved in the brains of mice fed persimmon leaf extracts containing quercetin, kaempferol and kaempferol derivatives [60]. Water-soluble dietary fiber, carotenoids and polyphenols derived from persimmons improved the lipid metabolism in male Wistar rats fed diets containing cholesterol [61]. In particular, tannins of persimmons containing gallocatechin, epigallocatechin-3- O-gallate and epicatechin-3-O-gallate have excellent antioxidant activity, such as with the 2-deoxyribose oxidation system and salicylic acid system, superoxide anion scavenging activity and linoleic acid lipid peroxidation inhibition activity [62]. Based on previous studies, the various physiologically active compounds contained in IPEE help to protect the Aβ-induced antioxidant system damage by significantly increasing the SOD and reduced GSH contents. In addition, IPEE inhibited the brain lipid peroxidation by inhibiting the oxidation of unsaturated fatty acids abundantly contained in the brain tissue. Thus, the consumption of persimmons, which have various polyphenols with excellent antioxidant activity, significantly reduced the antioxidant system deficits and might be an ingredient that can ameliorate the amyloidogenic cognitive function.

Aβ impairs the mitochondrial function by blocking mitochondrial translocation through damage to nuclear coding proteins such as electron transport chain components [63]. In addition, oligomeric Aβ easily enters organelles such as the mitochondria through the translocase of the outer mitochondrial membrane (TOM) complex through the lipid bilayer [64]. Aβ absorbed into the mitochondria complexly affects the function of electron transfer and promotes the apoptosis pathway by impairment of the mitochondrial dynamics and calcium storage [65]. In addition, Aβ interacts with the mitochondrial matrix components, such as the enzymes of the Krebs cycle containing pyruvate dehydrogenase (PDH), ATP-citrate lyase and acetoacetyl-CoA thiolase. Intramitochondrial Aβ peptides damage mitochondrial DNA and reduce ATP production with the reduction of the mitochondrial membrane potential [66]. Therefore, this study confirmed the ameliorating effect on mitochondrial function in Aβ-induced mice (Figure 3). According to a previous study, immature persimmon fruits contain a variety of tannins, such as catechin, catechin-3-gallate, gallocatechin and gallocatechin-3-gallate [67]. Catechin polyphenols reach the brain even in small amounts and have a protective effect on electron transport chains by reducing the mitochondrial damage of ROS caused by aging [68]. In particular, EGCG contained in persimmons improves the mitochondrial function by increasing the activity of mitochondrial cytochrome c oxidase (complex IV) and ATP synthase in neurons and astrocytes [69]. In addition, persimmons contain a large amount of quercetin, and the treatment with quercetin decreased the ROS and increased the ATP levels in the hippocampal mitochondria through increased AMP-activated protein kinase (AMPK) activity [70]. IPEE showed a mitochondrial-protective effect by significantly eliminating the ROS content inside the mitochondria. In addition, through the mitochondrial-protective effect, IPEE increases the survival of neuronal cells in brain tissue by improving the mitochondrial membrane potential and ATP production, affecting the energy supply. In conclusion, IPEE had a protective effect on mitochondrial deficits by improving the energy metabolism in neuronal cells.

Excessively produced Aβ stimulates the levels of inflammatory factors such as TNF in microglial cells, and increased TNF promotes the phosphorylation of JNK in neuronal cells [71]. The increase in p-JNK decreases the level of p-Akt related to cell survival and increases the expression of p53 and p-NF-κB, resulting in an inflammatory response [72]. The decrease in the expression of p-Akt lowers the expression of p-GSK3β, affects the stabilization of microtubules and induces the phosphorylation of tau protein present in the microtubules [73]. This p-tau is constantly oligomerized and, ultimately, produces NFT, resulting in neuronal death [11]. In addition, an increased inflammatory response indicates an increase in the content of BAX and the release of cytochrome c, which induces a cascade of apoptosis through the increased expression of caspase 3 and caspase 9 [74]. In this study, the regulatory effect of IPEE was assessed based on these pathways, and IPEE suppressed the expression of TNF-α, p-JNK, p-tau, p-NF-κB, BAX and caspase 3 and increased the expression of p-Akt and p-GSK3β (Figure 5). According to a previous study, an ethyl acetate fraction from persimmons regulated the JNK/Akt pathway by suppressing the expression of TNF-α, p-JNK and IRS-1pSer in trimethyltin chloride (TMT)-induced mice [59]. Additionally, persimmon extract ameliorated the diabetic cognitive dysfunction by regulating the expression of p-JNK, p-Akt and p-AMPK in HFD-induced mice [46]. Ellagic acid contained in IPEE reduced the BAX/BCl-2 ratio and expression of caspase 3 in D-galactose-induced rats [75]. In addition, quercetin suppressed cognitive deficit dietary advanced glycation end product-induced aged mice by reducing cathepsin B, tau phosphorylation and neuroinflammation such as the glial fibrillary acidic protein (GFAP) and Ibα1 [76]. Similar to a previous study, IPEE increased the neuronal survival by regulating the expression of p-JNK, p-Akt and p-GSK3β through JNK/Akt pathway regulation. In addition, IPEE reduced the expression of inflammatory factors such as TNF-α, p-JNK and p-NF-κB through various pathways and significantly suppressed apoptosis by reducing the expression of BAX and caspase 3. Therefore, it showed a protective effect against the toxicity of the tau protein induced by this pathway. Therefore, IPEE ameliorated amyloidogenic cognitive dysfunction by regulating the expression of TNF-α, p-JNK, p-Akt, p-GSK3β, p-tau, p-NF-κB, BAX and caspase 3 related to the tau pathway.

## 5. Conclusions

In summary, the administration of IPEE suppressed the memory and behavioral deficit by regulating the antioxidant capacity, cholinergic system and mitochondrial function via the tau pathway and apoptosis signaling in Aβ_1–42_-induced ICR mice. In conclusion, IPEE, which is largely discarded during the harvest period, might be used as a functional food material with an anti-amnesic effect against amyloidogenic damage and can increase the industrial value and help solve economic and environmental problems. In the future, to confirm which compounds actually help improve cognitive function, we are planning to assess the changes in bioactive compounds of IPEE through digestion, absorption and pharmacokinetic and pharmacodynamic (PK/PD) analyses compared to mature persimmons.

## Figures and Tables

**Table 1:** Compounds identified from the immature persimmon ethyl acetate fraction (IPEE).

No	RT (min)	Parent Ion ^a^ (*m/z*)	MS^2^ Ion (*m/z*)	Compound
1	1.84	169	125	Gallic acid
2	3.19	301	283, 257, 229, 185	Ellagic acid
3	3.60	505	301	Quercetin 3-O-(6″-acetyl-glucoside)

^a^ Ions are presented as *m/z* [M-H]^−^.

## Data Availability

The data presented in this study are available on request from the corresponding author.

## References

[1] Glenner G.G. (1983). Alzheimer’s disease. The commonest form of amyloidosis. Arch. Pathol. Lab. Med..

[2] Munoz D.G., Feldman H. (2000). Causes of Alzheimer’s disease. Can. Med. Assoc. J..

[3] Selkoe D.J. (2002). Alzheimer’s disease is a synaptic failure. Science.

[4] Kayed R., Head E., Thompson J.L., McIntire T.M., Milton S.C., Cotman C.W., Glabe C.G. (2003). Common structure of soluble amyloid oligomers implies common mechanism of pathogenesis. Science.

[5] Hardy J., Selkoe D.J. (2002). The amyloid hypothesis of Alzheimer’s disease: Progress and problems on the road to therapeutics. Science.

[6] Fridovich I. (1986). Biological effects of the superoxide radical. Arch. Biochem. Biophys..

[7] Li N., Sioutas C., Cho A., Schmitz D., Misra C., Sempf J., Wang M., Oberley T., Froines J., Nel A. (2003). Ultrafine particulate pollutants induce oxidative stress and mitochondrial damage. Environ. Health Perspect..

[8] Chen Z., Zhong C. (2014). Oxidative stress in Alzheimer’s disease. Neurosci. Bull..

[9] McAlpine F.E., Lee J.K., Harms A.S., Ruhn K.A., Blurton-Jones M., Hong J., Das P., Golde T.E., LaFerla F.M., Oddo S. (2009). Inhibition of soluble TNF signaling in a mouse model of Alzheimer’s disease prevents pre-plaque amyloid-associated neuropathology. Neurobiol. Dis..

[10] Saito A., Suzuki H.I., Horie M., Ohshima M., Morishita Y., Abiko Y., Nagase T. (2013). An integrated expression profiling reveals target genes of TGF-β and TNF-α possibly mediated by microRNAs in lung cancer cells. PLoS ONE..

[11] Johnson G.V., Stoothoff W.H. (2004). Tau phosphorylation in neuronal cell function and dysfunction. J. Cell Sci..

[12] Kang W.W., Kim J.K., Oh S.L., Kim J.H., Han J.H., Yang J.M., Choi J.U. (2004). Physicochemical characteristics of Sangju traditional dried persimmons during drying process. J. Korean Soc. Food Sci. Nutr..

[13] Yokozawa T., Lee Y.A., Cho E.J., Matsumoto K., Park C.H., Shibahara N. (2011). Anti-aging effects of oligomeric proanthocyanidins isolated from persimmon fruits. Drug Discov. Ther..

[14] Lee Y.A., Cho E.J., Tanaka T., Yokozawa T. (2007). Inhibitory activities of proanthocyanidins from persimmon against oxidative stress and digestive enzymes related to diabetes. J. Nutr. Sci. Vitaminol..

[15] Jo K.J., Lee J.M., Lee S.C., Park H.R. (2011). Anticancer activity of persimmon (*Diospyros kaki* L.) calyx extracts on human cancer cells. J. Med. Plants Res..

[16] Gato N., Kadowaki A., Hashimoto N., Yokoyama S.I., Matsumoto K. (2013). Persimmon fruit tannin-rich fiber reduces cholesterol levels in humans. Ann. Nutr. Metab..

[17] Cia P., Benato E.A., Sigrist J.M., Sarantopóulos C., Oliveira L.M., Padula M. (2006). Modified atmosphere packaging for extending the storage life of ‘Fuyu’persimmon. Postharvest Biol. Technol..

[18] Jeon J.H., Lee S.Y., Lee J.M., Ji D.H., Oh C.J. (2015). The development and application of standard diagnostic table for astringent persimmon management. J. Korean For. Soc..

[19] Kim J.E., Nam J.H., Cho J.Y., Kim K.S., Hwang D.Y. (2017). Annual tendency of research papers used ICR mice as experimental animals in biomedical research fields. Lab. Anim. Res..

[20] Yoo S.K., Kim J.M., Park S.K., Kang J.Y., Han H.J., Park H.W., Kim C.W., Lee U., Heo H.J. (2019). Chemical compositions of different cultivars of astringent persimmon (*Diospyros kaki* thunb.) and the effects of maturity. J. Food Sci. Technol..

[21] Jeong E.J., Lee H.K., Lee K.Y., Jeon B.J., Kim D.H., Park J.H., Song J.H., Huh J.M., Lee J.H., Sung S.H. (2013). The effects of lignan-riched extract of Shisandra chinensis on amyloid-β-induced cognitive impairment and neurotoxicity in the cortex and hippocampus of mouse. J. Ethnopharmacol..

[22] Wang D.M., Yang Y.J., Zhang L., Zhang X., Guan F.F., Zhang L.F. (2013). Naringin enhances CaMKII activity and improves long-term memory in a mouse model of Alzheimer’s disease. Int. J. Mol. Sci..

[23] Cho N., Lee H.K., Jeon B.J., Kim H.W., Kim H.P., Lee J.H., Kim Y.C., Sung S.H. (2014). The effects of *Betula platyphylla* bark on amyloid beta-induced learning and memory impairment in mice. Food Chem. Toxicol..

[24] Kim J.M., Park S.K., Kang J.Y., Park S.B., Yoo S.K., Han H.J., Cho K.H., Kim J.C., Heo H.J. (2019). Green tea seed oil suppressed Aβ_1–42_-induced behavioral and cognitive deficit via the Aβ-related Akt pathway. Int. J. Mol. Sci..

[25] Van der Borght K., Havekes R., Bos T., Eggen B.J., Van der Zee E.A. (2007). Exercise improves memory acquisition and retrieval in the Y-maze task: Relationship with hippocampal neurogenesis. Behav. Neurosci..

[26] Newman J.P., Kosson D.S. (1986). Passive avoidance learning in psychopathic and nonpsychopathic offenders. J. Abnorm. Psychol..

[27] Morris R. (1984). Developments of a water-maze procedure for studying spatial learning in the rat. J. Neurosci. Methods..

[28] Bradford M.M. (1976). A rapid and sensitive method for the quantitation of microgram quantities of protein utilizing the principle of protein-dye binding. Anal. Biochem..

[29] Vincent D., Segonzac G., Vincent M.C. (1958). Colorimetric determination of acetylcholine by the Hestrin hydroxylamine reaction and its application in pharmacy. Ann. Pharm. Fr..

[30] Ellman G.L., Courtney K.D., Andres V., Featherstone R.M. (1961). A new and rapid colorimetric determination of acetylcholinesterase activity. Biochem. Pharmacol..

[31] Liu F., Ng T.B. (2000). Effect of pineal indoles on activities of the antioxidant defense enzymes superoxide dismutase, catalase, and glutathione reductase, and levels of reduced and oxidized glutathione in rat tissues. Biochem. Cell Biol..

[32] Kim D.O., Jeong S.W., Lee C.Y. (2003). Antioxidant capacity of phenolic phytochemicals from various cultivars of plums. Food Chem..

[33] Crimins J.L., Pooler A., Polydoro M., Luebke J.I., Spires-Jones T.L. (2013). The intersection of amyloid beta and tau in glutamatergic synaptic dysfunction and collapse in Alzheimer’s disease. Ageing Res. Rev..

[34] Kása P., Rakonczay Z., Gulya K. (1997). The cholinergic system in Alzheimer’s disease. Prog. Neurobiol..

[35] Hampel H., Mesulam M.M., Cuello A.C., Farlow M.R., Giacobini E., Grossberg G.T., Khachaturian Z.S. (2018). The cholinergic system in the pathophysiology and treatment of Alzheimer’s disease. Brain.

[36] Kaminsky Y.G., Kosenko E.A. (2008). Effects of amyloid-beta peptides on hydrogen peroxide-metabolizing enzymes in rat brain in vivo. Free Radic. Res..

[37] Murphy M.P., LeVine H. (2010). Alzheimer’s disease and the amyloid-β peptide. J. Alzheimers Dis..

[38] Zhang C., Rissman R.A., Feng J. (2015). Characterization of ATP alternations in an Alzheimer’s disease transgenic mouse model. J. Alzheimers Dis..

[39] Wang X., Wang W., Li L., Perry G., Lee H.G., Zhu X. (2014). Oxidative stress and mitochondrial dysfunction in Alzheimer’s disease. Biochim. Biophys. Acta-Mol. Basis Dis..

[40] James J., Fiji N., Roy D., MG D.A., Shihabudeen M.S., Chattopadhyay D., Thirumurugan K. (2015). A rapid method to assess reactive oxygen species in yeast using H 2 DCF-DA. Anal. Methods.

[41] Butterfield D.A., Drake J., Pocernich C., Castegna A. (2001). Evidence of oxidative damage in Alzheimer’s disease brain: Central role for amyloid β-peptide. Trends Mol. Med..

[42] Behl C., Moosmann B. (2002). Oxidative nerve cell death in Alzheimers disease and stroke: Antioxidants as neuroprotective compounds. Biol. Chem..

[43] Butterfield D.A. (2002). Amyloid β-peptide (1-42)-induced oxidative stress and neurotoxicity: Implications for neurodegeneration in Alzheimer’s disease brain. A review. Free Radic. Res..

[44] Akiyama H., Barger S., Barnum S., Bradt B., Bauer J., Cole G.M., Wyss–Coray T. (2000). Inflammation and Alzheimer’s disease. Neurobiol. Aging..

[45] Das L., Bhaumik E., Raychaudhuri U., Chakraborty R. (2012). Role of nutraceuticals in human health. J. Food Sci. Technol..

[46] Shin E.J., Kim J.M., Kang J.Y., Park S.K., Han H.J., Kim H.J., Kim C.W., Lee U., Heo H.J. (2021). Ameliorative effect of persimmon (*Diospyros kaki*) in cognitively impaired diabetic mice. J. Food Biochem..

[47] Kaur R., Parveen S., Mehan S., Khanna D., Kalra S. (2015). Neuroprotective effect of ellagic acid against chronically scopolamine induced Alzheimer’s type memory and cognitive dysfunctions: Possible behavioural and biochemical evidences. Int. J. Preven. Med. Res..

[48] Yokozawa T., Park C.H., Noh J.S., Roh S.S. (2014). Role of oligomeric proanthocyanidins derived from an extract of persimmon fruits in the oxidative stress-related aging process. Molecules.

[49] Pákáski M., Kálmán J. (2008). Interactions between the amyloid and cholinergic mechanisms in Alzheimer’s disease. Neurochem. Int..

[50] Reyes A.E., Chacón M.A., Dinamarca M.C., Cerpa W., Morgan C., Inestrosa N.C. (2004). Acetylcholinesterase-Aβ complexes are more toxic than Aβ fibrils in rat hippocampus: Effect on rat β-amyloid aggregation, laminin expression, reactive astrocytosis, and neuronal cell loss. Am. J. Pathol..

[51] Jeong D.W., Cho C.H., Lee J.S., Lee S.H., Kim T., Kim D.O. (2018). Deastringent peel extracts of persimmon (*Diospyros kaki* Thunb. cv. Cheongdo-Bansi) protect neuronal PC-12 and SH-SY5Y cells against oxidative stress. J. Microbiol. Biotechnol..

[52] Mansouri M.T., Naghizadeh B., Ghorbanzadeh B., Farbood Y., Sarkaki A., Bavarsad K. (2013). Gallic acid prevents memory deficits and oxidative stress induced by intracerebroventricular injection of streptozotocin in rats. Pharmacol. Biochem. Behav..

[53] Hajipour S., Sarkaki A., Farbood Y., Eidi A., Mortazavi P., Valizadeh Z. (2016). Effect of gallic acid on dementia type of Alzheimer disease in rats: Electrophysiological and histological studies. Basic Clin. Neurosci..

[54] Christen Y. (2000). Oxidative stress and Alzheimer disease. Am. J. Clin. Nutr..

[55] Blasko I., Veerhuis R., Stampfer-Kountchev M., Saurwein-Teissl M., Eikelenboom P., Grubeck-Loebenstein B. (2000). Costimulatory effects of interferon-γ and interleukin-1β or tumor necrosis factor α on the synthesis of Aβ1-40 and Aβ1-42 by human astrocytes. Neurobiol. Dis..

[56] Jhoo J.H., Kim H.C., Nabeshima T., Yamada K., Shin E.J., Jhoo W.K., Kim W., Kang K.S., Joe S.A., Woo J.I. (2004). β-Amyloid (1–42)-induced learning and memory deficits in mice: Involvement of oxidative burdens in the hippocampus and cerebral cortex. Behav. Brain Res..

[57] Love S. (1999). Oxidative stress in brain ischemia. Brain Pathol..

[58] Coyle J.T., Puttfarcken P. (1993). Oxidative stress, glutamate, and neurodegenerative disorders. Science.

[59] Kim J.M., Park S.K., Kang J.Y., Park S.B., Yoo S.K., Han H.J., Kim C.W., Lee U., Kim S.H., Heo H.J. (2018). Ethyl acetate fraction from persimmon (*Diospyros kaki*) ameliorates cerebral neuronal loss and cognitive deficit via the JNK/Akt pathway in TMT-induced mice. Int. J. Mol. Sci..

[60] Huang S.W., Wang W., Zhang M.Y., Liu Q.B., Luo S.Y., Peng Y., Bei S., De-Ling W., Song S.J. (2016). The effect of ethyl acetate extract from persimmon leaves on Alzheimer’s disease and its underlying mechanism. Phytomedicine.

[61] Gorinstein S., Bartnikowska E., Kulasek G., Zemser M., Trakhtenberg S. (1998). Dietary persimmon improves lipid metabolism in rats fed diets containing cholesterol. J. Nutr..

[62] Gu H.F., Li C.M., Xu Y.J., Hu W.F., Chen M.H., Wan Q.H. (2008). Structural features and antioxidant activity of tannin from persimmon pulp. Food Res. Int..

[63] Devi L., Prabhu B.M., Galati D.F., Avadhani N.G., Anandatheerthavarada H.K. (2006). Accumulation of amyloid precursor protein in the mitochondrial import channels of human Alzheimer’s disease brain is associated with mitochondrial dysfunction. J. Neurosci..

[64] Alikhani N., Ankarcrona M., Glaser E. (2009). Mitochondria and Alzheimer’s disease: Amyloid-β peptide uptake and degradation by the presequence protease, hPreP. J. Bioenerg. Biomembr..

[65] Mark R.J., Pang Z., Geddes J.W., Uchida K., Mattson M.P. (1997). Amyloid β-peptide impairs glucose transport in hippocampal and cortical neurons: Involvement of membrane lipid peroxidation. J. Neurosci..

[66] Pagani L., Eckert A. (2011). Amyloid-Beta interaction with mitochondria. Int. J. Alzheimers Dis..

[67] Matsuo T., Ito S. (1978). The chemical structure of kaki-tannin from immature fruit of the persimmon (*Diospyros kaki* L.). Agric. Biol. Chem..

[68] Assuncao M., Andrade J.P. (2015). Protective action of green tea catechins in neuronal mitochondria during aging. Front. Biosci..

[69] Castellano-González G., Pichaud NBallard J.W.O., Bessede A., Marcal H., Guillemin G.J. (2016). Epigallocatechin-3-gallate induces oxidative phosphorylation by activating cytochrome c oxidase in human cultured neurons and astrocytes. Oncotarget.

[70] Wang D.M., Li S.Q., Wu W.L., Zhu X.Y., Wang Y., Yuan H.Y. (2014). Effects of long-term treatment with quercetin on cognition and mitochondrial function in a mouse model of Alzheimer’s disease. Neurochem. Res..

[71] Vukic V., Callaghan D., Walker D., Lue L.F., Liu Q.Y., Couraud P.O., Romero I.A., Weksler B., Stanimirovic D.B., Zhang W. (2009). Expression of inflammatory genes induced by beta-amyloid peptides in human brain endothelial cells and in Alzheimer’s brain is mediated by the JNK-AP1 signaling pathway. Neurobiol. Dis..

[72] Cabal-Hierro L., Rodríguez M., Artime N., Iglesias J., Ugarte L., Prado M.A., Lazo P.S. (2014). TRAF-mediated modulation of NF-kB AND JNK activation by TNFR2. Cell. Signal..

[73] Yu Y., Run X., Liang Z., Li Y., Liu F., Liu Y., Iqbal K., Grundke-Iqbal I., Gong C.X. (2009). Developmental regulation of tau phosphorylation, tau kinases, and tau phosphatases. J. Neurochem..

[74] Clementi M.E., Pezzotti M., Orsini F., Sampaolese B., Mezzogori D., Grassi C., Giardina B., Misiti F. (2006). Alzheimer’s amyloid β-peptide (1–42) induces cell death in human neuroblastoma via bax/bcl-2 ratio increase: An intriguing role for methionine 35. Biochem. Biophys. Res. Commun..

[75] Chen P., Chen F., Zhou B. (2018). Antioxidative, anti-inflammatory and anti-apoptotic effects of ellagic acid in liver and brain of rats treated by D-galactose. Sci. Rep..

[76] Yang S., Zhou H., Wang G., Zhong X.H., Shen Q.L., Zhang X.J., Li R.Y., Chen L.H., Zhang Y.H., Wan Z. (2020). Quercetin is protective against short-term dietary advanced glycation end products intake induced cognitive dysfunction in aged ICR mice. J. Food Biochem..

